# Supplementary material for: Disruptions of Circadian Genes in Cutaneous Melanoma—An In Silico Analysis of Transcriptome Databases
Source: Int J Mol Sci. 2023 Jun 14;24(12):10140. doi: 10.3390/ijms241210140 (PMC10299421; doi:10.3390/ijms241210140)
Supplement: Supplementary file 1 [file ijms-24-10140-s001.zip › ijms-2395946-supplementary.pdf]

Supplemental Table S1. Core circadian genes selected for the in siclico analysis

L.p.	Gene	Gene name Entrez	Gene ID	ID of transcript	Function	
**1**	*NR1D1*	Nuclear receptor subfamily 1 group d member 1	9572	NM_021724.5	The transcription factors, a members of the nuclear receptor subfamily 1 specifically the NR1 subfamily of receptors. The encoded protein is a ligand-sensitive transcription factor that negatively regulates the expression of core clock proteins. In particular this protein represses the BMAL1	https://www.genecards.org/cgi-bin/carddisp.pl?gene=NR1D1
**2**	*NR1D2*	Nuclear receptor subfamily 1 group d member 2	9975	NM_001145425.2		https://www.genecards.org/cgi-bin/carddisp.pl?gene=NR1D2
**3**	*DBP*	D-box binding PAR bZIP transcription factor	1628	NM_001352. 5	A member of the PAR bZIP transcription factor family and binds to specific sequences in the promoters of several gene bind DNA as a homo- or heterodimer and is involved in the regulation of some circadian rhythm gene.	https://www.genecards.org/cgi-bin/carddisp.pl?gene=DBP
**4**	*TEF*	Tef, par bzip transcription factor	7008	NM_001145398.3	A member of the PAR (proline and acidic amino acid-rich) subfamily of basic region/leucine zipper (bZIP) transcription factors.	https://www.genecards.org/cgi-bin/carddisp.pl?gene=TEF
**5**	*HLF*	Par bzip transcription factor	3131	NM_001330375.2	A member of the proline and acidic-rich (PAR) protein family, a subset of the bZIP transcription factors	https://www.genecards.org/cgi-bin/carddisp.pl?gene=HLF
**6**	*BHLHE40*	Basic helix-loop-helix family member e40	8553	NM_003670.3	A basic helix-loop-helix protein interact with ARNTL or compete for E-box binding sites in the promoter of PER1 and repress CLOCK/ARNTL's transactivation of PER1.	https://www.genecards.org/cgi-bin/carddisp.pl?gene=BHLHE40
**7**	*BHLHE41*	Basic helix-loop-helix family member e41	79365	NM_030762.3	A basic helix-loop-helix protein expressed in various tissues. The encoded protein can interact with ARNTL or compete for E-box binding sites in the promoter of PER1 and repress CLOCK/ARNTL's transactivation of PER1. This gene is believed to be involved in the control of circadian rhythm and cell differentiation.	https://www.genecards.org/cgi-bin/carddisp.pl?gene=BHLHE41

Supplemental Table S1. Core circadian genes selected for the in siclico analysis

**8**	*PER1*	Period circadian clock 1	5187	NM_002616.3	Period family of genes and is expressed in a circadian pattern in the suprachiasmatic nucleus, the primary circadian pacemaker in the mammalian brain. Genes in this family encode components of the circadian rhythms of locomotor activity, metabolism, and behavior. This gene is upregulated by CLOCK/ARNTL heterodimers but then represses this upregulation in a feedback loop using PER/CRY heterodimers to interact with CLOCK/ARNTL	https://www.genecards.org/cgi-bin/carddisp.pl?gene=PER1
**9**	*PER2*	Period circadian clock 2	8864	NM_022817.3		https://www.genecards.org/cgi-bin/carddisp.pl?gene=PER2
**10**	*PER3*	Period circadian clock 3	8863	NM_001289861.2		https://www.genecards.org/cgi-bin/carddisp.pl?gene=PER3
**11**	*CRY1*	Cryptochrome circadian clock 1	1407	NM_004075.5	A flavin adenine dinucleotide-binding protein that is a key component of the circadian core oscillator complex, which regulates the circadian clock. This gene is upregulated by CLOCK/ARNTL heterodimers but then represses this upregulation in a feedback loop using PER/CRY heterodimers to interact with CLOCK/ARNTL.	https://www.genecards.org/cgi-bin/carddisp.pl?gene=CRY1
**12**	*CRY2*	Cryptochrome circadian clock 2	1408	NM_001127457.3		https://www.genecards.org/cgi-bin/carddisp.pl?gene=CRY2
**13**	*RORA*	Rar related orphan receptor a	6095	NM_002943.4	The members of the NR1 subfamily of nuclear hormone receptors. It can bind as a monomer or as a homodimer to hormone response elements upstream of several genes to enhance the expression of those genes.	https://www.genecards.org/cgi-bin/carddisp.pl?gene=RORA
**14**	*RORB*	Rar related orphan receptor b	6096	NM_006914.34		https://www.genecards.org/cgi-bin/carddisp.pl?gene=RORB
**15**	*RORC*	Rar related orphan receptor c	6097	NM_001001523.2		https://www.genecards.org/cgi-bin/carddisp.pl?gene=RORC

Supplemental Table S1. Core circadian genes selected for the in siclico analysis

**16**	*NFIL3*	Nuclear factor, interleukin 3 regulated	4783	NM_001289999.2	A transcriptional regulator that binds as a homodimer to activating transcription factor (ATF) sites in many cellular and viral promoters. The encoded protein represses PER1 and PER2 expression and therefore plays a role in the regulation of circadian rhythm.	https://www.genecards.org/cgi-bin/carddisp.pl?gene=NFIL3
**17**	*ARNTL /BMAL1*	Aryl hydrocarbon receptor nuclear translocator like	406	NM_001030272.3	A basic helix-loop-helix protein that forms a heterodimer with CLOCK. This heterodimer binds E-box enhancer elements upstream of Period (PER1, PER2, PER3) and Cryptochrome (CRY1, CRY2) genes and activates transcription of these genes. PER and CRY proteins heterodimerize and repress their own transcription by interacting in a feedback loop with CLOCK/ARNTL complexes.	https://www.genecards.org/cgi-bin/carddisp.pl?gene=BMAL1
**18**	*CLOCK*	Clock circadian regulator	9575	NM_001289826.2	A transcription factor of the basic helix-loop-helix (bHLH) family and contains DNA binding histone acetyltransferase activity<br>The encoded protein forms a heterodimer with ARNTL (BMAL1) that binds E-box enhancer elements upstream of Period (PER1, PER2, PER3) and Cryptochrome (CRY1, CRY2) genes and activates transcription of these genes.	https://www.genecards.org/cgi-bin/carddisp.pl?gene=CLOCK
**19**	*NPAS2*	Neuronal pas domain protein 2	4862	NM_002518.4	A member of the basic helix-loop-helix (bHLH)-PAS family of transcription factors	https://www.genecards.org/cgi-bin/carddisp.pl?gene=NPAS2
**20**	*CIPC*	Clock interacting pacemake	85457	NM_033426.3	A negative regulator of circadian rhythm and negative regulation of transcription, DNA-templated.	https://www.genecards.org/cgi-bin/carddisp.pl?gene=CIPC
**21**	*CSNK1A1L*	Casein kinase 1 alpha 1 like	12011	NM_145203.6	A protein serine/threonine kinase activity<br>transferase activity, transferring phosphorus-containing groups and protein tyrosine kinase activity. An important paralog of this gene is CSNK1A1.	https://www.genecards.org/cgi-bin/carddisp.pl?gene=CSNK1A1L

Supplemental Table S1. Core circadian genes selected for the in siclico analysis

**22**	*CSNK1A1*	Casein kinase 1 alpha 1	1452	NM_001025105.3		https://www.genecards.org/cgi-bin/carddisp.pl?gene=CSNK1A1
**23**	*CSNK1E*	Casein kinase 1 epsilon	1454	NM_001894.6	Aserine/threonine protein kinase and a member of the casein kinase I protein family, whose members have been implicated in the control of cytoplasmic and nuclear processes, including DNA replication and repair.	https://www.genecards.org/cgi-bin/carddisp.pl?gene=CSNK1E
**24**	*TIMELESS*	Timeless circadian regulator	8914	NM_001330295.3	The protein encoded by this gene is highly conserved and is involved in cell survival after damage or stress, increase in DNA polymerase epsilon activity, maintenance of telomere length, and epithelial cell morphogenesis. The encoded protein also plays a role in the circadian rhythm autoregulatory loop, interacting with the PERIOD genes (PER1, PER2, and PER3) and others to downregulate activation of PER1 by CLOCK/ARNTL.	https://www.genecards.org/cgi-bin/carddisp.pl?gene=TIMELESS
